# Efficacy of patching combined with action video games in amblyopic children aged 4–10 years: A randomised clinical trial

**DOI:** 10.1111/opo.13534

**Published:** 2025-06-06

**Authors:** Laura Asensio‐Jurado, Marc Argilés, Valldeflors Vinuela‐Navarro, Lluïsa Quevedo‐Junyent, Dennis M. Levi

**Affiliations:** ^1^ Centre for Sensors, Instruments and Systems Development (CD6) Terrassa Spain; ^2^ School of Optics and Optometry Universitat Politècnica de Catalunya Terrassa Spain; ^3^ Hospital Universitari Mútua Terrassa Terrassa Spain; ^4^ Vision Optometry and Health (VOS) Terrassa Spain; ^5^ Herbert Wertheim School of Optometry and Vision Science University of California, Berkeley Berkeley California USA

**Keywords:** action video game, amblyopia, anisometropia, occlusion, strabismus

## Abstract

**Purpose:**

To determine the impact of using child‐friendly action video games combined with monocular occlusion for the treatment of children with amblyopia compared with occlusion alone.

**Methods:**

Twenty‐eight children aged 4–10 years (mean age: 5.80 ± 1.54 years) with anisometropic and/or strabismic amblyopia were included. Each participant was previously prescribed an optimal refractive correction, and after 8 weeks was randomly assigned to one of the treatment groups: action video games (AVG) plus occlusion (*n* = 14) or passive occlusion alone (PO) (*n* = 14). Visual acuity (VA) and stereoacuity (ST) were measured at baseline and following 14, 28 and 42 h of treatment. Compliance was monitored using parental registry and Google Analytics.

**Results:**

After 42 h, both groups showed significant improvement in the visual acuity of the amblyopic eye, *p* < 0.001 and *p* = 0.04 for the AVG and PO groups, respectively. However, VA recovery was significantly greater (*p* = 0.01) and faster with monocular AVG plus occlusion (0.18 logMAR) compared with occlusion alone (0.06 logMAR) in the amblyopic eye. The small improvement (0.02 logMAR) in the non‐amblyopic eye was not significant for either group. No significant differences between groups were observed for ST (*p* = 0.38).

**Conclusions:**

These findings suggest that combining occlusion and action video games at home results in greater effectiveness and efficiency in improving VA in children with anisometropic and strabismic amblyopia.

**Key points**

- Combining action video games with patching improved vision in children with amblyopia more effectively than patching alone, given equal treatment time.
- Binocular recovery is promoted through active treatments such as combining occlusion with the use of action video games.
- This home‐based video game treatment is accessible, requires no special equipment and is well accepted by families, with high adherence and engagement among children.

## INTRODUCTION

Amblyopia is an important developmental condition that affects up to 2.9% of the population.^1^ The standard treatment in childhood remains refractive correction, if appropriate, followed by occlusion treatment or optical penalisation. Despite the proven efficacy of this gold standard treatment, the visual function of many children does not reach normal levels, even after long periods of treatment.^2^, ^3^, ^4^, ^5^, ^6^ In fact, an average improvement in visual acuity (VA) of 0.10 logMAR has been observed after ≈ 120 h of occlusion.^6^, ^7^ In addition, there is evidence for the limitations of this treatment in terms of non‐response^6^, ^7^ or non‐compliance,^8^, ^9^ and up to 25% of patients experience a recurrence during the first year.^8^ These limitations underscore the need for alternative strategies that are both effective and more engaging. This study explores whether the combination of action video games with occlusion therapy can enhance treatment efficacy and adherence in children with amblyopia.

In recent years, there has been a growing interest in developing more efficient therapies for amblyopia, using binocular treatment and perceptual learning techniques.^10^, ^11^, ^12^, ^13^ Moreover, playing action video games (AVG) has been reported to produce significant improvements in various visual functions in normal adults,^14^, ^15^, ^16^, ^17^, ^18^ including spatial and temporal resolution and contrast sensitivity.^15^, ^16^, ^19^ Enhancements have also been documented in certain cognitive aspects, such as memory, attention and some aspects of executive function.^10^, ^20^, ^21^, ^22^, ^23^, ^24^, ^25^, ^26^, ^27^, ^28^

Li et al. found that playing AVG induced visual system plasticity. Their experiment, in a small group of adults with amblyopia, showed that playing AVG for 40–80 hours resulted in substantial improvement in a wide range of visual functions, including visual acuity (VA), spatial attention and stereopsis (ST).^29^

The present study aimed to compare monocular home‐based AVG treatment with conventional occlusion therapy in terms of efficacy and compliance in anisometropic and/or strabismic amblyopic children aged 4–10 years.

## METHODS

The aim of this study was to compare the effectiveness of monocular treatment using AVG on monocular VA and ST compared with conventional occlusion treatment in children with amblyopia who had not undergone amblyopia treatment previously. In addition, the compliance and degree of acceptance of the participants and their families were assessed.

### Study design

This study was a multicentre, randomised and prospective clinical trial in which child participants with refractive anisometropic and/or strabismic amblyopia were recruited from September 2019 to December 2022. Written informed consent was obtained from the parents or guardians of the study participants. The Clinical Research Ethics Committee of the Hospital Universitari Mútua de Terrassa (EO‐INT‐1910) and Fundació Sant Joan de Déu (PIC‐73‐19) approved the research protocol. The study was conducted in accordance with the Guide to Good Clinical Practices for Clinical Trials and conformed to the precepts and guidelines of the Declaration of Helsinki on the ethical principles of biomedical research in humans.

### Participant selection, randomisation and baseline measurements

Thirty children aged 4–10 years were evaluated and recruited by the Optometry department of the Hospital Universitari Mútua de Terrassa and Parc Universitari Sant Joan de Déu. The randomisation sequences were generated in a 1:1 ratio using IBM SPSS statistical software (version 25, ibm.com) by a researcher who was independent of the study.

Eligible participants were children diagnosed with amblyopia of refractive and/or strabismic origin, with an interocular VA difference ≥0.20 logMAR^30^, ^31^ and a manifest deviation ≤20 prism dioptres (Δ). Only participants without associated ophthalmological pathology were included. Exclusion criteria comprised any prior or current amblyopia treatment, bilateral amblyopia, deprivation amblyopia or the presence of ocular media opacities (e.g., corneal opacities, cataract), nystagmus, ptosis, retinal disease or optic nerve pathology.

Participants were classified into two groups based on the aetiology of their amblyopia: that is, anisometropic or strabismic. Anisometropic amblyopia was defined as a refractive difference between the two eyes >1.5 dioptres spherical equivalent or 1.0 dioptre of astigmatism, in the absence of a manifest ocular deviation. Individuals were classified as strabismic amblyopes if they exhibited a constant manifest ocular deviation in at least one position of gaze. Participants with mixed characteristics were classified in the strabismic amblyopia group.

All participants underwent a comprehensive visual examination, and refractive error correction was prescribed if needed. During the following 8 weeks, participants were able to adapt to the refractive correction.^32^

### Treatment protocols

**FIGURE 1:**
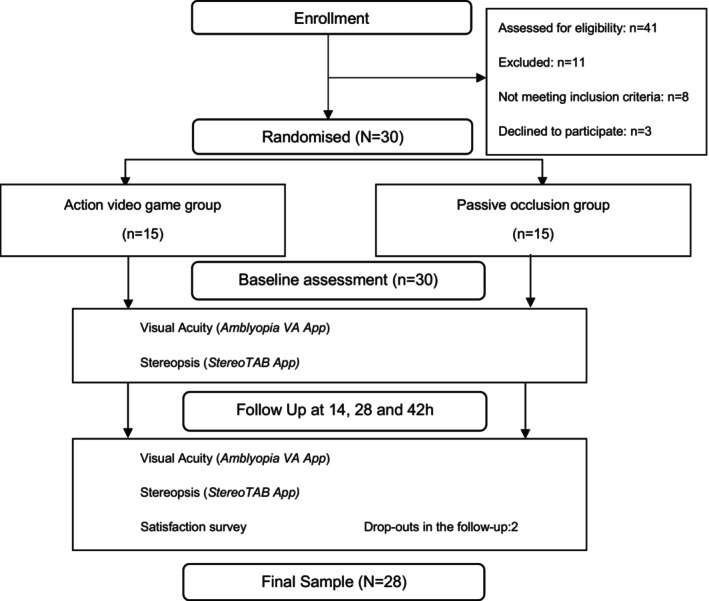
Consolidated standards of reporting trials (CONSORT) diagram of the study.

Children were randomly assigned to either the AVG group or the passive occlusion (PO) group. The AVG group underwent monocular occlusion of the non‐amblyopic eye combined with playing AVG 1 h a day for 42 days (42 h/42 sessions). The PO group underwent 2 h of daily occlusion of the non‐amblyopic eye (42 h/21 sessions) (Figure 1). Both treatments were performed at home and the instructions provided to the subjects in each group are detailed in Data S2.

The selection of video games was based on elements that characterise AVG,^21^, ^33^ but with the commitment to being non‐violent and child‐friendly. Participants accessed the selected video games using either a computer or tablet through a customised website created for the study (ambliopiatt.cat) and at a viewing distance of approximately 40 cm. The video games used for the treatment and their characteristics are detailed in the supplementary information. Participants in the PO group were instructed to engage in any daily activities while wearing the patch, excluding the use of mobile phones, tablets or computers for gaming. During follow‐up visits, participants or their families were asked about the activities performed while patching.

At the end of the intervention, the participants and their parents completed a questionnaire to assess the degree of satisfaction with the treatment and the impact on the quality of life of the participants and their families (Data S3 and S4).

### Outcome measures

VA and ST were evaluated using an iPad and a series of apps. The computer tools used were AmblyopiaVA and StereoTAB (Indaloftal SL, qvisionacademy.com) (Data S5).^34^, ^35^

Best‐corrected visual acuity (BCVA) was measured at 2 m with the Snellen E optotype of the AmblyopiaVA App. This was designed in accordance with the amblyopia treatment study protocol for children under 6 years of age,^36^ with the measurements being made in 0.10 LogMAR steps. ST measurements were made using StereoTAB (Indaloftal SL, qvisionacademy.com), a random dot test measured at 50 cm with green/red glasses.^34^ In addition, the degree of acceptance, satisfaction and adherence to the treatment was assessed for each participant.

### Adherence

Adherence to the treatment protocol was assessed using a daily log recorded by the parents in both treatment groups. In addition, in the AVG group, compliance with the prescribed hours was monitored using Google Analytics (developers.google.com/analytics).

After the random allocation of participants to the AVG group, each received a link to access the website containing the selected video games. The link was unique for every participant, and using the Google Analytics tool, the research team was able to assign an individual metric to each participant, to assess their activity on the video game platform and the number of hours completed in accordance with the study.

At the end of the treatment, the adherence of the participants was assessed as follows: good compliance ≥75% of the prescribed treatment completed; moderate compliance, 60%–74% and poor compliance being <60% of the prescribed treatment completed.

### Statistical methods

To assess differences between the two treatment groups (AVG and PO) and the two subject populations (anisometropic or strabismic amblyopia), analyses focused on performance improvement over time. Because the two tests (VA and ST) were on different scales, the measures were first converted to logarithmic notation for analysis.

The first analysis was a repeated measures multivariate analysis of variance (ANOVA). The dependent variables were VA and ST. Therefore, ANOVA was run with the within‐subject factors of time (four levels, i.e., baseline, 14, 28 and 42 h), the between factors of treatment type (AVG and PO) and the amblyopia type (anisometropic or strabismus). Greenhouse–Geisser or Huynh–Feldt correction was used for the model.

## RESULTS

Overall, 41 children were screened between September 2019 and October 2022. Thirty were randomised and 28 were treated and their data analysed. The reasons for exclusion and study discontinuation are shown in Figure 1. Participants' mean age ± SD was 5.80 ± 1.54 years and 15 were female (53.57%). There were no statistically significant differences between the groups, including mean age (*p* = 0.37) at baseline. Fourteen participants were included in the AVG group with nine anisometropic amblyopes and five strabismic amblyopes, and 14 participants were included in the PO group with nine anisometropic amblyopes and five strabismic amblyopes. Baseline demographics and characteristics are shown in Table S1.

**FIGURE 2:**
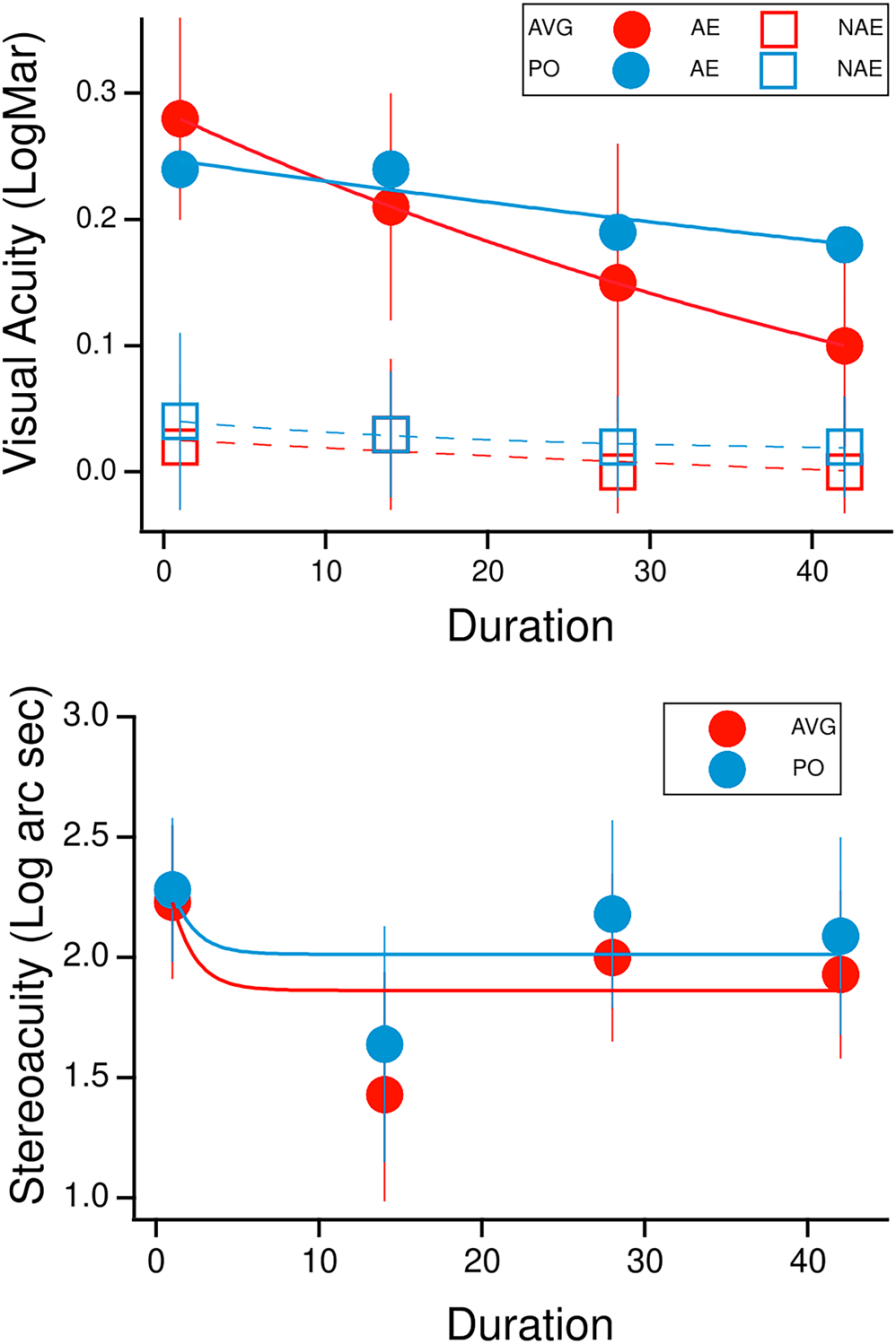
(Top). Change in visual acuity (logMAR) in the amblyopic eye (AE) and non‐amblyopic eye (NAE) and (bottom) change in stereoacuity (log arc sec) in the AVG group (red) and PO group (blue) before treatment and 14, 28 and 42 h after the intervention. The curves are exponential fits to the data. AVG, action video games plus occlusion; PO, passive occlusion alone.

The study set out to determine whether combining AVG play with occlusion therapy was more effective than PO in 4‐ to 10‐year‐old children with anisometropic and/or strabismic amblyopia. The results (Figure 2 Top) indicated that while both groups showed a significant improvement in VA in the amblyopic eye (*F*(3, 39)=57.23, *p* < 0.001, η_p_^2^ = 0.82 and *F*(3, 39)=6.69, *p* = 0.04, η_p_^2^ = 0.34 in the AVG and PO groups, respectively), the improvement in VA with the amblyopic eye was significantly greater in the AVG (0.18 logMAR) than in the PO group (0.06 logMAR) (*F*(3, 78) = 6.87, *p* = 0.01, η_p_^2^ = 0.21). Post hoc comparisons (Bonferroni‐adjusted) identified significant differences between time points, notably between the initial VA and after 42 h of treatment (*p* < 0.001), reflecting steady progress in VA. The small improvement (0.02 logMAR) in VA observed in the non‐amblyopic eye was not significant for either group, AVG (*p* = 0.48) and PO (*p* = 0.32) or between groups (*F*(1.88, 48.9) = 0.81, *p* = 0.45, η_*p*_^2^ = 0.03). In order to compare the time course for the improvement of the amblyopic eye, exponential curves were fitted to the data shown in Figure 2. The mean time constants (number of hours to reach 1/*e*) were 65.6 ± 9 for the amblyopic eye of the AVG group and 154.6 ± 3 for the amblyopic eye of the PO group. These very different time constants suggest that improvement is more rapid when AVG play is combined with patching.

There was no significant difference in VA between the two groups prior to treatment (*p* = 0.46). For the amblyopic eye, the initial mean BCVA ± SD for the AVG and PO groups was 0.28 ± 0.08 logMAR and 0.24 ± 0.11 logMAR, respectively. In the non‐amblyopic eye, VA in the AVG and PO groups was 0.02 ± 0.06 logMAR and 0.04 ± 0.07 logMAR, respectively. Upon completion of the intervention, VA in the amblyopic eye of the AVG and PO groups was 0.10 ± 0.08 logMAR and 0.18 ± 0.09 logMAR (*p* < 0.001 and *p* = 0.04), respectively. The respective values for the non‐amblyopic eye were 0.00 ± 0.06 logMAR and 0.02 ± 0.04 logMAR (*p* = 0.48 and *p* = 0.33).

The VA results are summarised in Table 1 and Figure 2.

**TABLE 1:** Descriptive statistics (mean ± standard deviation) for visual acuity (VA) in the two groups (expressed in logMAR units).

	VA pre	VA 14 h	VA 28 h	VA 42 h	*p*‐Value
AVG	NAE = 0.02 ± 0.05	NAE = 0.03 ± 0.06	NAE = 0.00 ± 0.06	NAE = 0.00 ± 0.06	0.48
(*n* = 14)	AE = 0.28 ± 0.08	AE = 0.21 ± 0.09	AE = 0.15 ± 0.11	AE = 0.10 ± 0.08	<0.001
PO	NAE = 0.04 ± 0.07	NAE = 0.03 ± 0.05	NAE = 0.02 ± 0.04	NAE = 0.02 ± 0.04	0.33
(*n* = 14)	AE = 0.24 ± 0.12	AE = 0.24 ± 0.08	AE = 0.19 ± 0.07	AE = 0.18 ± 0.09	0.04

*Note*: *p*‐Values are from the repeated measures ANOVA.

Abbreviations: AE, amblyopic eye; AVG, action video games plus occlusion; NAE, non‐amblyopic eye; PO, passive occlusion alone.

In regard to possible differences between anisometropic (*n* = 18) and strabismic (*n* = 10) amblyopes, repeated measures ANOVA indicated a significant difference (*F*(3, 48) = 10.42, *p* < 0.001, η_p_^2^ = 0.39) in regard to the improvement in VA for the amblyopic eye of the anisometropic participants, with a greater improvement in the AVG group. No significant differences were observed between the treatment groups in the strabismic amblyopes (*F*(3, 24) = 0.57, *p* = 0.63, η_p_^2^ = 0.07). No significant difference in VA improvement was found between treatments in the non‐amblyopic eye of the anisometropic and strabismic subgroups, *F*(1.56, 24.96) = 0.97, *p* = 0.38, η_p_^2^ = 0.07 and *F*(3, 24)=2.00, *p* = 0.14, η_p_^2^ = 0.20, respectively.

Mean ST (Figure 2, bottom and Table 2) showed a significant improvement from baseline after 42 h of treatment in both the AVG and the PO groups, *F*(3, 39) = 56.21, *p* < 0.001, η_p_^2^ = 0.81 and *F*(3, 39)=22.75, *p* < 0.001, η_p_^2^ = 0.64, respectively. No significant effect of time × group was observed between groups (*F*(2, 13)=55.60, *p* = 0.38, η_p_^2^ = 0.37). Post hoc comparisons (Bonferroni‐adjusted) showed significant differences between specific time points, particularly between the initial and 14 h ST (*p* < 0.001), as well as ST at 14 and 28 h (*p* < 0.001), suggesting an initial improvement followed by decline, with no significant differences between 28 and 42 h (*p* = 0.29). This can be seen in Figure 2 which shows the ST results of both groups. Most of the improvement occurred rapidly between baseline and 14 h (time constants were 1.4 h for both groups) with no further improvement.

**TABLE 2:** Descriptive statistics (mean ± standard deviation) of stereoacuity (ST) in log arc sec in the two groups.

	ST pre	ST 14 h	ST 28 h	ST 42 h	*p*‐Value
AVG (*n* = 14)	2.23 ± 0.32	1.43 ± 0.51	2.00 ± 0.35	1.93 ± 0.35	<0.001
PO (*n* = 14)	2.28 ± 0.30	1.64 ± 0.49	2.18 ± 0.39	2.09 ± 0.41	<0.001

*Note*: *p*‐Values are from the repeated measures ANOVA.

Abbreviations: AVG, action video games plus occlusion; PO, passive occlusion alone.

In regard to whether the treatment modality was effective depending on if the amblyopia was strabismic or anisometropic, no significant differences in stereoacuity improvement between treatments (AVG or PO) were found for either anisometropic, *F*(1.92, 30.68) = 0.63, *p* = 0.53, η_p_^2^ = 0.04 or strabismic amblyopia, *F*(1.45, 11.62) = 1.65, *p* = 0.23, η_p_^2^ = 0.17. Although the results did not reach statistical significance, the observed trend suggested that participants with anisometropic amblyopia tended to respond better to treatment compared with strabismic amblyopes, *F*(3, 36)=0.84, *p* = 0.48, η_p_^2^ = 0.07 in the AVG group and *F*(3, 36)=2.66, *p* = 0.06, η_p_^2^ = 0.18 in the PO group. This is supported by the effect sizes, which indicated a moderate to large proportion of variance explained by the anisometropic group's improvement with both treatments.

A linear regression analysis was performed, treating severity as a continuous variable. In particular, the relationship between VA and ST was examined, as well as their respective improvements following treatment. This approach sought to investigate whether baseline severity was associated with the magnitude of improvement.

For the AVG group, baseline VA did not predict improvement in VA in the amblyopic eye (*r* = 0.20, *p* = 0.35), explaining only 7.35% of the variance. Baseline ST explained 50.76% of the variance for the improvement in ST (*r* = 0.41, *p* = 0.004), indicating that better baseline ST predicted greater improvement.

**FIGURE 3:**
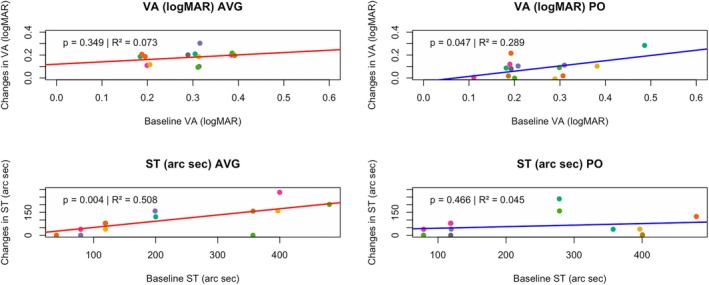
(Top) Linear regression analyses examining the relationship between baseline visual acuity (VA) and the improvement in VA following treatment. (Bottom) Linear regression analyses examining the relationship between baseline stereoacuity (ST) and improvement in ST following treatment. The left panels show the results from the action video games plus occlusion (AVG) group, while the right panels show the results from the passive occlusion only (PO) group. The corresponding *p*‐values and *R* ^2^ values are displayed on each graph.

In the PO group, baseline VA also showed no significant association with the improvement in VA in the amblyopic eye (*r* = −0.39, *p* = 0.81). Also, the relationship between baseline ST and improvement in ST was not significant (*r* = 0.10, *p* = 0.47) (Figure 3).

There was no significant relationship between the improvement in VA in the amblyopic eye and interocular VA in either group. For the AVG and PO groups, *r*‐values were 0.13 (*p* = 0.57) and −2.31 (*p* = 0.49), respectively.

Twenty‐eight participants (93%) completed the 42‐h visit (Figure 1). Mean compliance was 36.01 ± 3.09 h (85.74%) and 38.43 ± 2.25 h (91.50%) in the AVG group, as analysed with Google Analytics tool and parent registration, respectively. In the PO group, the mean (SD) compliance was 39.66 ± 2.36 h (94.42%) (Table S2). When comparing Google Analytics monitoring versus parental control, ANOVA revealed a significant difference in compliance percentages between groups, *F*(1, 26) = 12.34, *p* = 0.001, η_p_^2^ = 0.32, indicating a moderate to large effect size. Post hoc Tukey HSD testing confirmed that the PO group had significantly better compliance than the AVG group. When compliance was monitored using parental control in both groups (AVG and PO), ANOVA did not find significant differences between groups, *F*(1, 26) = 1.99, *p* = 0.17, η_p_^2^ = 0.07, suggesting a small effect size. Tukey HSD testing also confirmed no significant differences between the groups. Compliance percentages were not significantly associated with improvements in VA or ST for either monitoring condition (Table S3).

## DISCUSSION

In this randomised clinical trial, following 42 h of treatment, VA improved more in participants playing AVG with monocular occlusion at home for 1 h/day (improvement of 0.18 logMAR or 51.36%), compared with PO for 2 h/day (improvement of 0.06 logMAR or 14.81%), and the time course of improvement was more rapid in the AVG group. Singh et al. evaluated the role of monocular video games as an adjunct to occlusion therapy in the treatment of anisometropic amblyopia in children aged 6–14 years, showing significantly greater improvements in the 1 h daily video game group (0.10 logMAR; 25.89% improvement) compared with the 6‐h occlusion‐only group (0.05 logMAR; 12.20% improvement) after 1 month of treatment.^37^ Furthermore, Li et al.^29^ showed that mean VA improved by 0.16 logMAR (44.54%) after 40 h of monocular AVG play in adults, in addition to displaying improvements in spatial attention and ST, while Vedamurthy et al. reported that after 40 h of therapy, average VA improved by 0.14 logMAR (38.03%) in their gaming dichoptic group.^18^

The present results align with those of Hernández‐Andrés et al.,^38^ who observed greater improvements in VA when patching was combined with active monocular interventions. VA gains were 0.29 logMAR for vision therapy and patching, 0.21 logMAR for perceptual learning and patching and 0.11 logMAR for patching alone. Both the current study and that of Hernández‐Andrés et al.^38^ found significant within‐group improvements in ST. In the current study, ST improved by 0.30 and 0.19 log arcsec in the AVG and PO groups, respectively, while Hernández‐Andrés et al. reported comparable gains across groups, with no significant differences between treatments. Collectively, these findings support the use of engaging monocular interventions to enhance the effectiveness of conventional occlusion therapy.

The monocular intervention with AVG used here, performed for a total of 42 h, demonstrated results similar to Gambacorta et al.,^33^ who employed a binocular intervention using dichoptic video games. Their approach yielded a mean improvement in VA of 0.14 logMAR, with only half of the treatment duration adopted here (20 h). These findings suggest that while extended monocular treatment may result in significant improvements in VA, a shorter binocular approach may offer an equally effective or potentially more efficient pathway to improve visual function, highlighting the advantages of involving both eyes in the therapeutic process, particularly for enhancing ST.

In the current investigation, ST improved significantly in both treatment groups, with an average gain of 0.30 and 0.19 log arc sec in the AVG and PO groups, respectively, although the difference between the groups was not significant. This contrasts with Gambacorta et al.,^33^ who did not show significant improvements in ST with 0.07 log arcsec in the dichoptic group versus 0.06 log arcsec in the monocular group. However, other studies have reported significant improvements in ST with the AVG or watching movies.^18^, ^39^, ^40^, ^41^, ^42^, ^43^ Zhu et al.^43^ investigated the effectiveness of combining stereoscopic movies with traditional patching, demonstrating that this combined approach resulted in significantly greater improvements in both VA and ST compared with patching alone. Specifically, the group that received the combined intervention showed a significantly greater mean gain (improvement of 0.47 log arcsec) compared with the patching‐only group (improvement of 0.14 log arcsec). They also reported a mean improvement in VA of 0.17 logMAR in the group that combined three‐dimensional (3D) movies with penalisation of the non‐amblyopic eye, which is very similar to the mean improvement of 0.18 logMAR observed in the present study using a monocular approach with AVG. This suggests that while different modalities were used, both interventions—whether binocular with 3D movies or monocular with AVG—could potentially achieve comparable improvements in VA. The use of 3D movies represents a more passive approach to treatment, as the participant simply watches preselected visual content without active engagement or interaction. In contrast, treatment involving AVG, as used here, is inherently more dynamic and requires continuous active participation, which engages attentional and motor skills. This interactivity may enhance visual learning through repeated visual‐motor coordination and decision‐making, potentially contributing to a greater level of neural plasticity compared with passive viewing.

While other studies implemented approaches that promoted active and passive binocular stimulation (using dichoptic video games and 3D films, respectively), the present study focused on monocular AVG, providing an accessible intervention in the home and requiring no special display equipment. These studies agree that the interventions using AVG and movies have a greater effect on the VA of the amblyopic eye compared with patching alone; however, only some studies with dichoptic intervention achieved better improvements in ST compared with patching alone.^40^, ^42^, ^43^

The finding that baseline severity predicts the magnitude of improvement in the AVG group but not in the PO group, particularly for ST, suggests that monocular intensive treatment with AVG can exploit to a larger extent the recovery potential in participants with greater initial deficits. In the AVG group, poorer baseline stereopsis was significantly associated with greater improvements in ST (*p* = 0.004, *R* ^2^ = 0.51), indicating that those with a larger initial deficit benefit more from this treatment. In contrast, for the PO group, neither baseline ST nor baseline VA had significant predictive power over the improvement obtained (ST: *p* = 0.47, *R* ^2^ = 0.05; VA: *p* = 0.81, *R* ^2^ = 0.004).

The absence of a significant correlation between baseline severity and improvement for either VA or ST in the PO group suggests that patching, as a passive treatment lacking dynamic stimuli, provides a more uniform and limited improvement regardless of the initial severity. In contrast, the significant relationship found in the AVG group can be attributed to the nature of the treatment; monocular training with AVG provides a dynamic and demanding visual stimulus that requires active visual engagement. This active training process likely enhances perceptual and motor improvements, making the treatment particularly effective for participants with more pronounced initial deficits in ST.

The current study design differed from that of many traditional amblyopia trials by equalising the total treatment time between groups to isolate the effect of adding AVGs. This also allowed consideration of the burden of daily treatment, an important factor in family acceptance and adherence. Thus, the present results show that, even with reduced daily patching hours, adding AVGs improved treatment efficacy significantly.

The present study shows that monocular treatment in combination with AVG provides more effective results than PO, with good accessibility to be performed at home. This effectiveness is further supported by a large effect size (Cohen's *d* = 0.89) and the statistical power of the analysis was 95.3%, well above the conventional threshold of 80%,^44^ confirming the robustness and clinical relevance of the findings. Unlike other studies where treatments were carried out in the laboratory or clinic using dichoptic or perceptual learning methods, this study evaluated a home‐based AVG intervention combined with monocular occlusion. This is a simple treatment procedure that can be easily accessed and followed at home by amblyopic children and their families. This observation is verified by the high treatment compliance rates. Participant adherence to the different treatments was similar in both groups (Table S1). However, it is important to note that treatment adherence between groups was monitored differently. When relying solely on parental control data, no significant differences were observed between the groups. In contrast, when using Google Analytics data for the experimental group, significant differences emerged, with the control group showing improved adherence. These findings underscore the potential bias introduced by subjective monitoring methods, such as parental control, which often overestimate adherence.^23^, ^45^, ^46^ This highlights the importance of employing objective tools whenever possible to ensure accurate measurement.

Despite these methodological considerations, participant adherence to monocular AVG treatment was excellent in 92.9% of the group, and only one participant of 14 had moderate adherence. In the PO group, compliance was excellent in 100% of cases according to parental records. However, it must be considered that the treatment duration evaluated was 42 h, and it is known that adherence to occlusion decreases with time.^39^, ^47^

AVGs were initially defined as shooting games and were associated with violent content. Currently, there seems to be a consensus in defining AVG as those containing a number of key elements that facilitate plasticity, such as fast moving objects that require a motor response with time constraints, due to a heavy perceptual and motor load, the need to plan objectives and to alternate between selective attention of specific objects to a more global and generalised attention^21^ Thus, video games without violent content can still be considered AVG and therefore useful for the management of young amblyopic children. The video games selected and grouped on the website had these characteristics, in addition to their appropriateness for the age of the participants.

Video games allow constant experimentation in a task that requires a set of perceptual and attentional skills, interactivity, immediate feedback and autonomy on the part of the viewer. Furthermore, they allow the introduction of incentive elements associated with treatment in clinical practice, such as a reward system or performance graphs, with the aim of encouraging compliance. However, it should be noted that there are different genres of video games, and not all of them contain perceptual and cognitive elements that characterise action video games, and that seem to be effective in improving visual function.

Including AVG in the treatment of amblyopia is an interesting option considering that they incorporate a rich and immersive environment. In addition, their popularity among children, together with their easy accessibility, makes them an excellent tool to improve compliance rates during occlusion treatment.

### Limitations

In addition to the small sample size, there were other limitations in the current study. First, most participants still had to continue treatment after short‐term therapy of only 42 h. Therefore, whether the difference in VA improvement between the two groups is maintained over the long term remains uncertain. However, even in the short term, improved VA can enhance the participants' quality of life. Second, treatment compliance may not be as high in clinical practice because brief follow‐up visits would not be maintained, along with daily treatment recording, and this could affect the magnitude and degree of improvement in visual function. Third, the randomisation of this study was conducted 8 weeks after an optical correction was provided, but some children might have benefited from a longer period of refractive adaptation before further treatment. However, the 8‐week randomisation was in line with previous studies and recommendations.^3^, ^5^, ^48^ This fixed 8‐week period also allows a better study design for statistical and clinical comparisons. Finally, the study included only mild and moderate cases of amblyopia. Future investigations should consider stratifying participants based on the severity of their condition to augment an understanding of treatment efficacy at different levels of visual impairment.

## CONCLUSIONS

The current study indicated that both monocular treatment with AVG and PO are effective for children aged 4–10 years with anisometropic and/or strabismic amblyopia. Combining AVG with occlusion resulted in a significantly greater and more rapid improvement in VA in the amblyopic eye than conventional occlusion after 42 h of treatment.

These findings have important clinical implications, as they support the integration of engaging, home‐based monocular video game interventions into standard amblyopia treatment protocols. Such approaches could enhance treatment efficiency, improve adherence and potentially reduce dropout rates, thereby offering a viable alternative for improving visual outcomes in paediatric patients.

## AUTHOR CONTRIBUTIONS

**Laura Asensio‐Jurado:** Conceptualization (equal); data curation (lead); formal analysis (lead); investigation (lead); methodology (equal); project administration (lead); resources (lead); software (equal); validation (equal); visualization (lead); writing – original draft (lead); writing – review and editing (equal). **Marc Argilés:** Conceptualization (lead); data curation (supporting); formal analysis (supporting); investigation (supporting); methodology (lead); project administration (equal); supervision (lead); validation (equal); writing – original draft (supporting); writing – review and editing (equal). **Valldeflors Vinuela‐Navarro:** Validation (equal); visualization (supporting); writing – original draft (supporting); writing – review and editing (equal). **Lluïsa Quevedo‐Junyent:** Conceptualization (lead); data curation (supporting); formal analysis (supporting); investigation (supporting); methodology (lead); project administration (equal); supervision (equal); validation (equal); visualization (supporting); writing – original draft (supporting); writing – review and editing (supporting). **Dennis M. Levi:** Formal analysis (supporting); supervision (equal); validation (equal); visualization (supporting); writing – original draft (supporting); writing – review and editing (equal).

## FUNDING INFORMATION

Nothing to declare.

## CONFLICT OF INTEREST STATEMENT

The authors declare that no conflict of interest.

## PATIENT CONSENT STATEMENT

All participating children understood the study protocol and directly provided verbal consent before participation. Written informed consent was also obtained from their parents or legal guardians.

## CLINICAL TRIAL REGISTRATION

The study was officially registered at ClinicalTrials.gov. Identifier: NCT04313257 [03/08/2020].

## Supporting information


Table S1.

Data S1.

## Data Availability

The datasets used and analysed during the current study are available from the following OSF link: https://osf.io/3w7tp/?view_only=581674a5b2c941aaa49a5c7e33f488e9.
