# Supplementary material for: Efficacy of patching combined with action video games in amblyopic children aged 4–10 years: A randomised clinical trial
Source: Ophthalmic Physiol Opt. 2025 Jun 6;45(6):1389–98. doi: 10.1111/opo.13534 (PMC12357224; doi:10.1111/opo.13534)
Supplement: Supplementary file 1 — Table S1. [file OPO-45-1389-s001.docx]

**Supplementary Table**

**Table S1. Baseline demographics and characteristics.**

**Table S2. Patient Adherence to the Prescribed Treatment**

**Table S3. Patient Adherence, visual acuity and stereopsis.**

**Table S1. Baseline demographics and characteristics. Abbreviations:** G, Group: 1, AVG; 2, PO. Type of amblyopia: S, strabismic amblyopia; A, anisometropic amblyopia. S, Sex; F, female; M, male. A, Age. **Cover test (CT):** Exo-deviations are represented by negative values, and eso-deviations by positive values. **Amblyopic eye:** R, right; L, left.

| **#** | **G** | **T** | **S** | **A** | **Refractive error** | | **CT_Far_pre** | **CT_Near_pre** | **AE** | **VA_AE_pre** | **VA_AE_14** | **VA_AE_28** | **VA_AE_42** | **VA_FE_pre** | **VA_FE_14** | **VA_FE_28** | **VA_FE_42** | **ST_pre** | **ST_14** | **ST_28** | **ST_42** |
| --- | --- | --- | --- | --- | --- | --- | --- | --- | --- | --- | --- | --- | --- | --- | --- | --- | --- | --- | --- | --- | --- |
| S1 | 2 | S | F | 5 | R | 170 -1,75 +1,00 | 4 | 4 | L | 0,50 | 0,30 | 0,20 | 0,20 | 0,20 | 0,10 | 0,00 | 0,10 | 357 | 357 | 318 | 318 |
|  |  |  |  |  | L | 175 -1,75 +3,50 |  |  |  |  |  |  |  |  |  |  |  |  |  |  |  |
| S2 | 1 | A | F | 6 | R | 180 -4,50 +3,00 | 2 | -1 | R | 0,20 | 0,10 | 0,00 | 0,00 | -0,10 | 0,00 | -0,10 | -0,10 | 119 | 40 | 40 | 40 |
|  |  |  |  |  | L | 0,00 |  |  |  |  |  |  |  |  |  |  |  |  |  |  |  |
| S3 | 1 | A | M | 7 | R | 125 -0,50 +3,75 | 2 | 2 | L | 0,20 | 0,10 | 0,00 | 0,00 | 0,00 | 0,00 | -0,10 | -0,10 | 40 | 40 | 40 | 40 |
|  |  |  |  |  | L | 35 -0,50 +4,75 |  |  |  |  |  |  |  |  |  |  |  |  |  |  |  |
| S4 | 2 | S | F | 5 | R | 180 -2,00 +1,25 | -8 | -10 | R | 0,20 | 0,20 | 0,20 | 0,00 | 0,00 | 0,00 | 0,00 | 0,00 | 79 | 238 | 397 | 79 |
|  |  |  |  |  | L | 175 -1,00 |  |  |  |  |  |  |  |  |  |  |  |  |  |  |  |
| S5 | 1 | A | F | 6 | R | 180 -0,25 +1,25 | 0 | 0 | L | 0,30 | 0,30 | 0,30 | 0,20 | 0,00 | 0,00 | 0,00 | 0,00 | 79 | 79 | 79 | 79 |
|  |  |  |  |  | L | 90 -0,25 +3,25 |  |  |  |  |  |  |  |  |  |  |  |  |  |  |  |
| S6 | 2 | A | M | 5 | R | 10 -3,50 +1,00 | 0 | -6 | R | 0,20 | 0,10 | 0,10 | 0,10 | 0,00 | 0,00 | 0,00 | 0,00 | 119 | 40 | 40 | 40 |
|  |  |  |  |  | L | 170 -2,50 +1,00 |  |  |  |  |  |  |  |  |  |  |  |  |  |  |  |
| S7 | 2 | A | F | 7 | R | 0,00 | 0 | -6 | L | 0,10 | 0,10 | 0,10 | 0,10 | -0,10 | 0,00 | 0,00 | 0,00 | 79 | 79 | 40 | 40 |
|  |  |  |  |  | L | 170 -1,00 +3,00 |  |  |  |  |  |  |  |  |  |  |  |  |  |  |  |
| S8 | 1 | A | F | 5 | R | 10 -1,50 +2,50 | 0 | 0 | R | 0,20 | 0,20 | 0,10 | 0,10 | 0,00 | 0,00 | 0,00 | 0,00 | 79 | 79 | 79 | 40 |
|  |  |  |  |  | L | 170 -0,75 +1,50 |  |  |  |  |  |  |  |  |  |  |  |  |  |  |  |
| S9 | 1 | S | M | 4 | R | 10 -2,50 +6,50 | 6 | 6 | R | 0,30 | 0,20 | 0,20 | 0,20 | 0,10 | 0,10 | 0,10 | 0,10 | 357 | 357 | 357 | 357 |
|  |  |  |  |  | L | 160 -2,50 +6,50 |  |  |  |  |  |  |  |  |  |  |  |  |  |  |  |
| S10 | 2 | A | F | 7 | R | 15 -1,00 +0,50 | 0 | 0 | L | 0,20 | 0,20 | 0,20 | 0,20 | 0,00 | 0,00 | 0,00 | 0,00 | 79 | 79 | 79 | 79 |
|  |  |  |  |  | L | 155 -1,00 -0,25 |  |  |  |  |  |  |  |  |  |  |  |  |  |  |  |
| S11 | 1 | S | F | 5 | R | 180 -2,75 +4,50 | 2 | 2 | R | 0,30 | 0,20 | 0,10 | 0,10 | 0,10 | 0,20 | 0,10 | 0,10 | 397 | 397 | 238 | 238 |
|  |  |  |  |  | L | 175 -1,75 +4,50 |  |  |  |  |  |  |  |  |  |  |  |  |  |  |  |
| S12 | 1 | S | M | 4 | R | 90 -1,50 +6,00 | 6 | 8 | R | 0,40 | 0,30 | 0,30 | 0,20 | 0,10 | 0,10 | 0,10 | 0,10 | 357 | 278 | 278 | 199 |
|  |  |  |  |  | L | 85 -0,75 +5,00 |  |  |  |  |  |  |  |  |  |  |  |  |  |  |  |
| S13 | 2 | A | M | 6 | R | 10 -1,50 +5,00 | 0 | 0 | R | 0,30 | 0,30 | 0,30 | 0,30 | 0,00 | 0,00 | 0,00 | 0,00 | 400 | 400 | 400 | 400 |
|  |  |  |  |  | L | 160 -0,50 +0,75 |  |  |  |  |  |  |  |  |  |  |  |  |  |  |  |
| S14 | 2 | S | M | 6 | R | 155 -1,25 | -14 | -14 | R | 0,20 | 0,20 | 0,20 | 0,20 | 0,00 | 0,10 | 0,10 | 0,10 | 400 | 397 | 397 | 397 |
|  |  |  |  |  | L | 30 -0,50 +0,25 |  |  |  |  |  |  |  |  |  |  |  |  |  |  |  |
| S15 | 1 | A | F | 6 | R | 5 -1,00 +1,00 | 0 | -2 | L | 0,30 | 0,20 | 0,16 | 0,10 | 0,00 | 0,00 | 0,00 | 0,00 | 119 | 79 | 79 | 40 |
|  |  |  |  |  | L | 175 -1,25 +4,00 |  |  |  |  |  |  |  |  |  |  |  |  |  |  |  |
| S16 | 1 | A | F | 6 | R | 180 -0,75 +3,00 | 0 | -2 | R | 0,3 | 0,3 | 0,2 | 0,10 | 0,00 | 0,00 | 0,00 | 0,00 | 200 | 119 | 79 | 79 |
|  |  |  |  |  | L | 10 -0,75 +2,00 |  |  |  |  |  |  |  |  |  |  |  |  |  |  |  |
| S17 | 2 | A | M | 6 | R | 180 -1,75 +6,25 | 0 | -4 | R | 0,20 | 0,20 | 0,10 | 0,10 | 0,00 | 0,00 | 0,00 | 0,00 | 119 | 119 | 119 | 119 |
|  |  |  |  |  | L | 180 -1,75 +5,50 |  |  |  |  |  |  |  |  |  |  |  |  |  |  |  |
| S18 | 1 | A | F | 6 | R | 5 -5,00 +0,75 | 0 | -2 | R | 0,20 | 0,10 | 0,10 | 0,00 | 0,00 | 0,00 | 0,00 | 0,00 | 119 | 79 | 79 | 40 |
|  |  |  |  |  | L | 170 -4,50 +2,00 |  |  |  |  |  |  |  |  |  |  |  |  |  |  |  |
| S19 | 1 | A | M | 4 | R | 180 -3,50 +3,75 | 0 | 0 | R | 0,30 | 0,10 | 0,00 | 0,00 | 0,10 | 0,10 | 0,00 | 0,00 | 199 | 79 | 40 | 40 |
|  |  |  |  |  | L | 170 -3,75 +3,75 |  |  |  |  |  |  |  |  |  |  |  |  |  |  |  |
| S20 | 2 | A | F | 5 | R | 5,50 | 0 | -3 | L | 0,20 | 0,20 | 0,10 | 0,10 | 0,00 | 0,10 | 0,10 | 0,10 | 278 | 79 | 79 | 40 |
|  |  |  |  |  | L | 5,00 |  |  |  |  |  |  |  |  |  |  |  |  |  |  |  |
| S21 | 1 | S | F | 4 | R | 0,00 | 14 | 16 | L | 0,40 | 0,30 | 0,30 | 0,20 | 0,00 | 0,00 | 0,00 | 0,00 | 400 | 357 | 200 | 119 |
|  |  |  |  |  | L | 0,00 |  |  |  |  |  |  |  |  |  |  |  |  |  |  |  |
| S22 | 2 | S | M | 7 | R | 150 -0,25 +2,50 | -30 | -20 | R | 0,30 | 0,30 | 0,30 | 0,30 | 0,00 | 0,00 | 0,00 | 0,00 | 480 | 480 | 397 | 357 |
|  |  |  |  |  | L | 20 -0,75 |  |  |  |  |  |  |  |  |  |  |  |  |  |  |  |
| S23 | 1 | S | M | 7 | R | 160 -0,25 +4,00 | -16 | -10 | R | 0,40 | 0,40 | 0,20 | 0,20 | 0,00 | 0,00 | 0,00 | 0,00 | 480 | 480 | 278 | 278 |
|  |  |  |  |  | L | 20 -0,75 +1,00 |  |  |  |  |  |  |  |  |  |  |  |  |  |  |  |
| S24 | 2 | A | M | 4 | R | 2,00 | 0 | 0 | L | 0,30 | 0,30 | 0,20 | 0,20 | 0,10 | 0,10 | 0,10 | 0,10 | 119 | 79 | 79 | 79 |
|  |  |  |  |  | L | 170 -2,50 +4,50 |  |  |  |  |  |  |  |  |  |  |  |  |  |  |  |
| S25 | 2 | A | F | 6 | R | 170 -0,25 +5,00 | 0 | -4 | L | 0,20 | 0,20 | 0,20 | 0,10 | 0,00 | 0,00 | 0,00 | 0,00 | 119 | 119 | 79 | 40 |
|  |  |  |  |  | L | 175 -1,00+700 |  |  |  |  |  |  |  |  |  |  |  |  |  |  |  |
| S26 | 2 | A | M | 4 | R | 180 -0,75 +2,00 | 0 | 0 | L | 0,30 | 0,30 | 0,20 | 0,20 | 0,10 | 0,10 | 0,00 | 0,00 | 278 | 119 | 119 | 119 |
|  |  |  |  |  | L | 180 -4,00 +4,00 |  |  |  |  |  |  |  |  |  |  |  |  |  |  |  |
| S27 | 1 | A | F | 5 | R | 90 -1,00 +4,25 | 4 | 5 | R | 0,20 | 0,20 | 0,10 | 0,10 | 0,00 | 0,00 | 0,00 | 0,00 | 119 | 79 | 79 | 79 |
|  |  |  |  |  | L | 85 -1,25 +2,00 |  |  |  |  |  |  |  |  |  |  |  |  |  |  |  |
| S28 | 2 | S | M | 6 | R | 60 -2,00 +5,50 | 16 | 16 | L | 0,40 | 0,40 | 0,30 | 0,30 | 0,00 | 0,00 | 0,00 | 0,00 | 397 | 397 | 357 | 357 |
|  |  |  |  |  | L | 1,75 |  |  |  |  |  |  |  |  |  |  |  |  |  |  |  |

**Table S2. Patient Adherence to the Prescribed Treatment**

|  | **Parent registration** | | **Analytics registration** |
| --- | --- | --- | --- |
| **Compliance** | **PO Group** | **AVG Group** | |
| **Good (74%-100%)** | 14 (100%) | 14 (100%) | 13 (92.86%) |
| **Moderate (60%–74%)** | 0 | 0 | 1 (7.14%) |
| **Poor (0%-59%)** | 0 | 0 | 0 |

| **Group** | **1W** | **2W (14h)** | **3W** | **4W (28h)** | **5W** | **6W (42h)** | **TOTAL** | **% C** |
| --- | --- | --- | --- | --- | --- | --- | --- | --- |
| **AVG Group** |  |  |  |  |  |  |  |  |
| **Parent registration** | 6.74 ± 0.74 | 6.23 ± 0.99 | 6.60 ± 0.42 | 6.59 ± 0.48 | 6.36 ± 0.97 | 5.92 ± 1.27 | 38.43 ± 2.25 | 91.50% |
| **Analytic registration** | 6.68 ± 0.90 | 5.96 ± 1.21 | 6.05 ± 0.49 | 6.02 ± 0.51 | 5.60 ± 1.02 | 5.7 ± 1.14 | 36.01 ± 3.09 | 85.74% |
| **PO Group** | **-** | **2W (14h)** | **-** | **4W (28h)** | **-** | **6W (42h)** | **TOTAL** | **% C** |
| **Parent registration** |  | 13.56 ± 0.61 |  | 13.38 ± 0.59 |  | 12.82 ± 1.83 | 39.66 ± 2.36 | 94.42% |

**Table S3. Patient Adherence, visual acuity and stereopsis.**

| **PO GROUP** | | | | | |
| --- | --- | --- | --- | --- | --- |
|  | Hours | Correlation | 1.00 | -0.14 | -0.23 |
|  |  | Significance | **.** | 0.615 | 0.418 |
| ST | VA | Correlation | -0.38 | 1.00 | **.** |
|  |  | Significance | 0.197 | **.** | **.** |
| VA | ST | Correlation | -0.41 | **.** | 1.00 |
|  |  | Significance | 0.154 | **.** | . |

| **Control variables** | **Variables** | | Hours | VA | ST |
| --- | --- | --- | --- | --- | --- |
| **AVG GROUP** | | | | | |
|  | Hours | Correlation | 1.00 | -0.17 | -0.53 |
|  |  | Significance | **.** | 0.555 | 0.049 |
| ST | VA | Correlation | -0.20 | 1.00 | **.** |
|  |  | Significance | 0.510 | **.** | **.** |
| VA | ST | Correlation | -0.54 | **.** | 1.00 |
|  |  | Significance | 0.56 | **.** | . |

Correlation and Partial correlation between hours with treatment response for visual acuity and stereoacuity after 42h of treatment when we control for the effect of the other variable (VA or ST) for both groups.
