# Supplementary material for: Efficacy of patching combined with action video games in amblyopic children aged 4–10 years: A randomised clinical trial
Source: Ophthalmic Physiol Opt. 2025 Jun 6;45(6):1389–98. doi: 10.1111/opo.13534 (PMC12357224; doi:10.1111/opo.13534)
Supplement: Supplementary file 2 — Data S1. [file OPO-45-1389-s002.docx]

**Supplementary Methods**

**Methods S1. Eligibility Exclusion Criteria**

**Methods S2. Patient Instructions for Different Groups**

**Methods S3. Treatment acceptability questionnaire**

**Methods S4. Action video game group satisfaction questionnaire.**

**Methods S5. Description of the assessment App.**

**Methods S1. Eligibility Exclusion Criteria**

The inclusion criteria were:

(1) Etiology of amblyopia: refractive and/or strabismic,

(2) Interocular VA difference less than or equal to 0.4 log MAR

(3) Angle of deviation equal to or less than 20Δ and

(4) Absence of associated ophthalmological pathology.

The exclusion criteria were:

(1) current or previous amblyopia treatment

(2) bilateral amblyopia

(3) amblyopia due to deprivation

(4) opacity of the ocular media such as corneal opacities; cataract; nystagmus; ptosis; retinal pathologies or optic neuropathies.

**Methods S2. Patient Instructions for Different Groups**

Below are the detailed information and instructions given to participants and their families for both treatment groups:

**PO goup:**

The treatment that *(child's name)* must follow involves covering the *(right or left)* eye from Monday to Sunday for 2 hours a day. The total duration of the treatment will be 21 days (3 weeks). During these hours, the child can engage in any activity (watching TV, reading, drawing, etc.) except using a mobile phone, tablet, or computer for playing games.

**AVG group:**

The treatment that *(child's name)* must follow involves covering the non-amblyopic eye for 1 hour a day for 42 days (6 weeks). In addition to the treatment time, the child should play certain video games, specifically those found on the website www.ambliopiatt.cat. The time spent on each video game is at the participant's discretion, although we recommend that it be as varied as possible. The video games have been selected based on very strict criteria of non-violence, and the prescribed usage time is limited and, therefore, lower than the recommendations.

The treatment involves covering the *(right or left)* eye and should be done from Monday to Sunday for 1 hour a day. This hour of treatment can be divided throughout the day, but into a maximum of 3 periods of 20 minutes each.

**Methods S3. Treatment acceptability questionnaire**

Questionnaire used in this study to evaluate the patient satisfaction.

| **Question** | **Answer** |
| --- | --- |
| Indicate the degree of confidence you have in the treatment followed to treat amblyopia. | 1-5 scale^a^ |
| Does this treatment have any side effects? | Yes / No |
| Indicate the degree of discomfort of the side effects of the treatment. | 1-5 scale^b^ |
| How much does the treatment interfere with your daily routine? | 1-5 scale^b^ |
| How difficult is it to carry out the treatment? | 1-5 scale^c^ |
| How difficult is it to access this treatment? | 1-5 scale^c^ |
| How satisfied are you with this treatment? | 1-5 scale ^d^ |
| How satisfied do you think the study patient is with the treatment? | 1-5 scale ^d^ |
| Indicate the degree of satisfaction with the time the treatment takes. | 1-5 scale ^d^ |

^a^ Confidence in the treatment was rated on a scale of 1 to 5: 1 = no confidence; 2 = Little confident; 3 = moderately confident; 4 = confident; 5 = very confident.

^b^ The degree to which an issue was addressed was assessed on a scale of 1 to 5: 1 = not at all; 2 = little; 3 = moderately; 4 = a lot; 5 = very much.

^c^ Difficulty of doing activities was rated on a scale of 1 to 5: 1 = not difficult; 2 = slight; 3 = moderate; 4 = difficult; 5 = inability.

^d^ Patient satisfaction was rated on a scale of 1 to 5: 1 = very satisfied; 2 = satisfied; 3 = neither satisfied nor dissatisfied; 4 = dissatisfied; 5 = very dissatisfied.

**Methods S4. Action video game group satisfaction questionnaire.**

| **Question** | **Answer** |
| --- | --- |
| Did you like the games? | Yes / No |
| Indicate the degree to which you liked the different games. |  |
| The Pirate Ship | 1-5 scale^a^ |
| The Saloon | 1-5 scale^a^ |
| Hooligans | 1-5 scale^a^ |
| Space Invanders | 1-5 scale^a^ |
| Yeti Sensation | 1-5 scale^a^ |
| Pizza Ninja | 1-5 scale^a^ |
| Relic Runway | 1-5 scale^a^ |
| Katana Fruits | 1-5 scale^a^ |
| Do you think it was easy to do this treatment? | Yes / No |
| Are there any games you found difficult? | Yes / No |
| Which ones? |  |

^a^ The degree to which an issue was addressed was assessed on a scale of 1 to 5: 1 = not at all; 2 = little; 3 = moderately; 4 = a lot; 5 = very much.

**Methods S5. Description of the assessment App.**

Visual acuity and stereoacuity will be assessed by means of an Ipad and a series of apps. Visual acuity will be evaluated with the AmbliopiaVA and stereopsis with the StereoTAB. Here is a summary of its functionality (see Rodríguez-Vallejo et al., 2017, Rodríguez-Vallejo, Monsoriu & Furlan, 2016, Rodríguez-Vallejo et al.,2016 for more information):

These applications are designed to facilitate accurate and detailed evaluation of different aspects of visual function, aiding eye care professionals in diagnosing and treating various eye conditions.

**AmblyopiaVA** is an application designed to assess visual acuity in patients with amblyopia. The app is intended for both clinical settings and home use, providing an accurate and accessible evaluation of visual acuity. Assesses visual acuity by presenting letters, numbers, or symbols that the patient must identify.

Displays a simple interface with various options to select the type of visual acuity test. The test adjusts the size of these characters to determine the smallest level the patient can correctly recognize. Presents different symbols or letters on the screen that the patient must recognize. The size of the symbols gradually decreases to determine the smallest level the patient can correctly identify. At the end of the test, the app provides a precise measure of the patient's visual acuity, typically in terms of Snellen lines or logMAR. Used to monitor the progression of amblyopia and evaluate the effectiveness of treatments such as patching or vision therapy. The app allows modifications in viewing distance and test difficulty, making it adaptable to different ages and severity levels of the condition. AmblyopiaVA provides visual acuity measurements in standardized formats such as Snellen lines or logMAR, which are easily interpretable by eye care professionals.

StereoTAB (**Fig 1**) is an application designed to assess stereopsis, or depth perception, which is the ability to perceive the world in three dimensions and judge the distances between objects. Stereopsis depends on the binocular integration of images from both eyes.

Displays stereoscopic images that the patient must view through special lenses (anaglyph or polarized). The patient identifies shapes or patterns, and the app measures the minimum angular disparity they can detect. Used to diagnose and evaluate conditions affecting binocular vision, such as strabismus, amblyopia, and convergence issues. Allows adjustments in viewing distance and type of stereopsis test (e.g., Random Dot, TNO) to meet different clinical needs. StereoTAB provides a precise measure of stereopsis in terms of arcminutes (arcmin) or arcseconds (arcsec), crucial for evaluating the patient's depth perception ability.


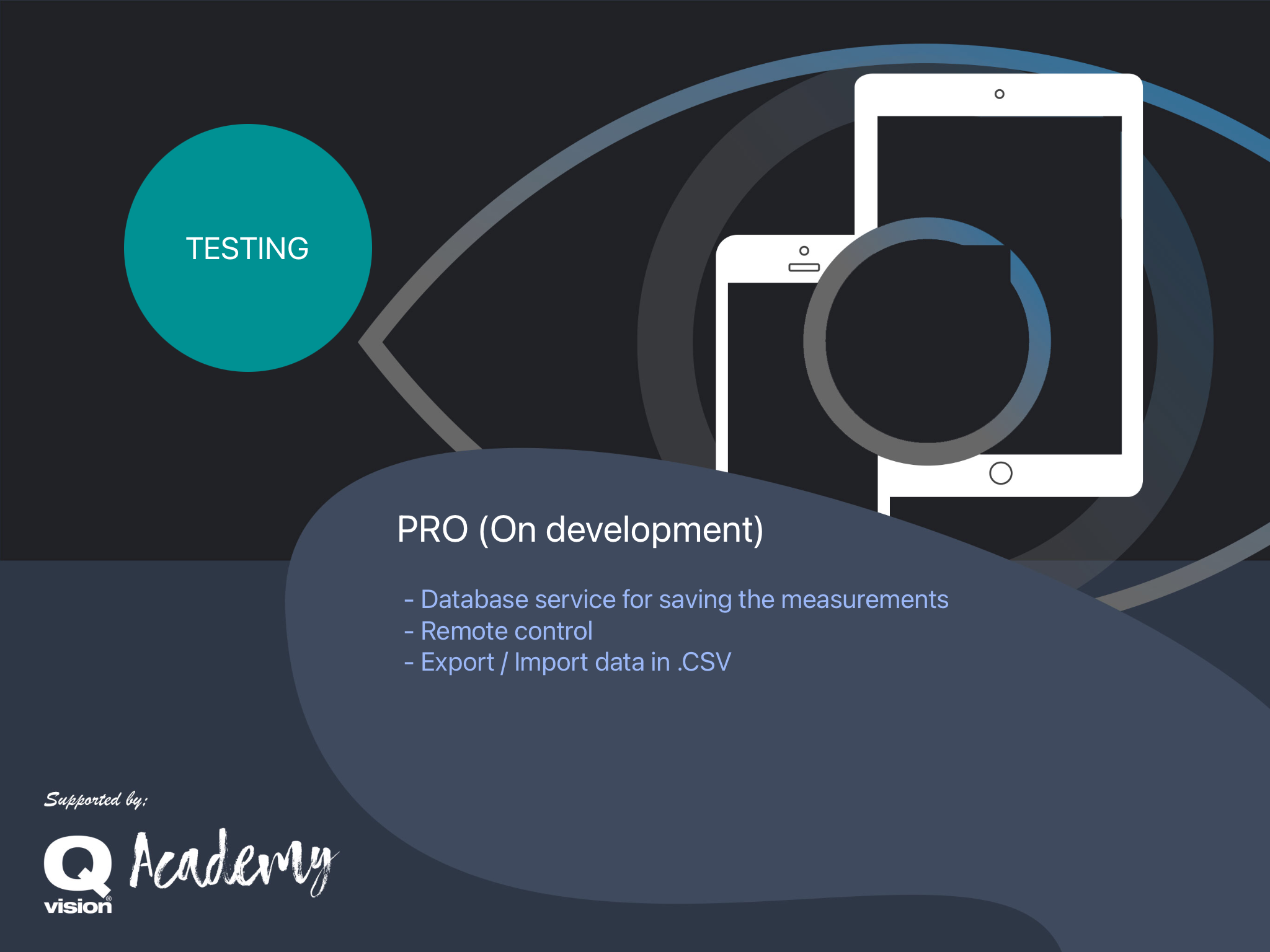

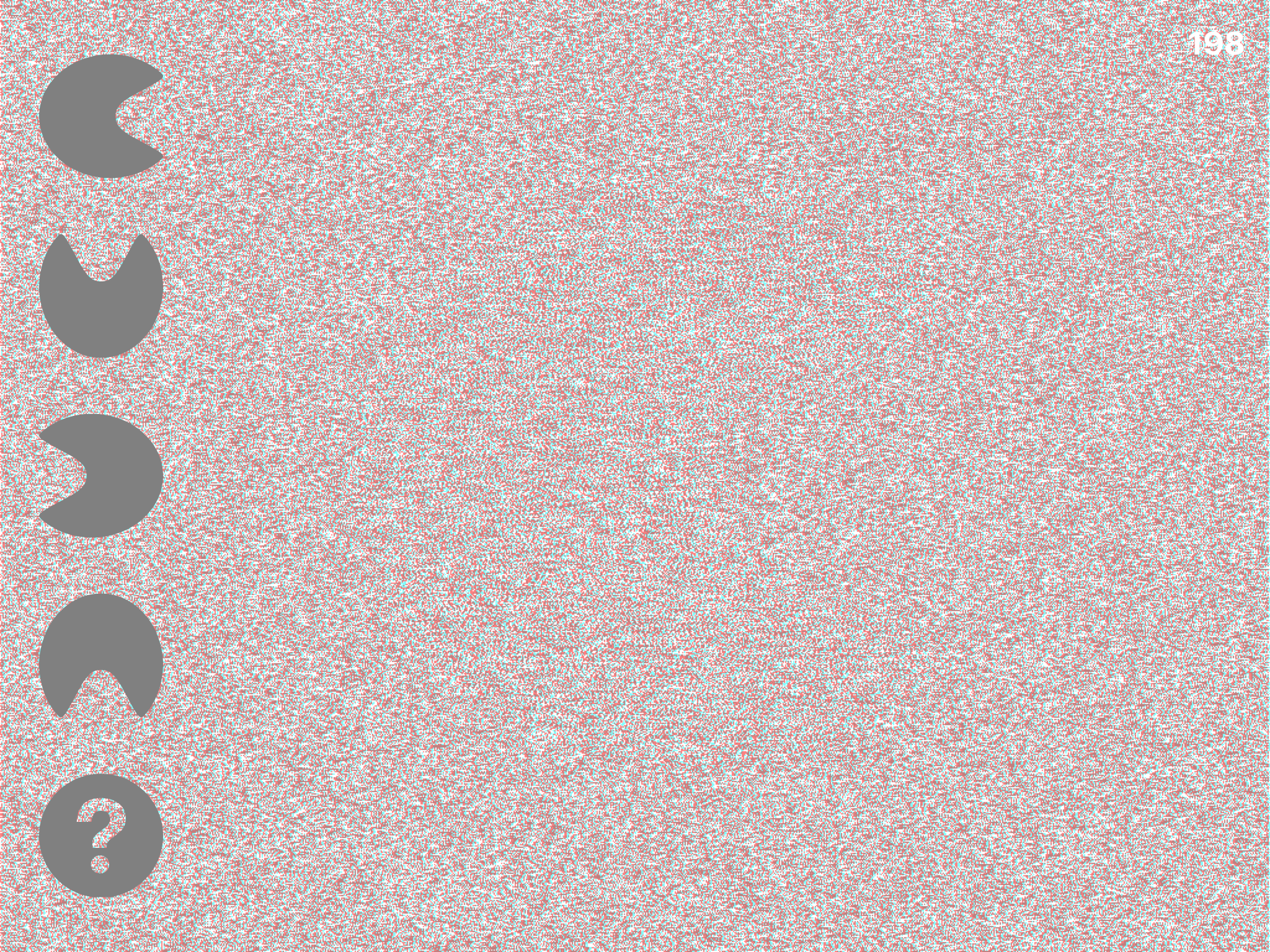


Fig 1. StereoTAB
